# Relationship between somatostatin receptor expressing tumour volume and health‐related quality of life in patients with metastatic GEP‐NET

**DOI:** 10.1111/jne.13139

**Published:** 2022-04-29

**Authors:** Håkan Ohlsson, Anni Gålne, Elin Trägårdh, Marlene Malmström, Anna Sundlöv, Martin Almquist

**Affiliations:** ^1^ Department of Surgery Ystad Hospital Ystad Sweden; ^2^ Institute of Clinical Sciences Lund University Lund Sweden; ^3^ Department of Medical Imaging and Physiology Skåne University Hospital Lund Sweden; ^4^ Department of Translational Medicine Lund University Malmö Sweden; ^5^ Department of Translational Medicine and Wallenberg Centre for Molecular Medicine Lund University Malmö Sweden; ^6^ Department of Surgery and Gastroenterology Skåne University Hospital Lund Sweden; ^7^ Department of Health Sciences Lund University Lund Sweden; ^8^ Division of Oncology Skåne University Hospital Lund Sweden; ^9^ Department of Clinical Sciences Lund University Lund Sweden; ^10^ Endocrine‐Sarcoma Unit, Department of Surgery Skåne University Hospital Lund Sweden

**Keywords:** health‐related quality of life, neuroendocrine tumours, somatostatin receptor imaging, tumour volume

## Abstract

For patients with gastroenteropancreatic neuroendocrine tumours (GEP‐NET), health‐related quality of life (HRQoL) is important. Meanwhile, whether tumour volume is associated with HRQoL is unknown. Hence, the aim of this study was to assess if total somatostatin receptor expressing tumour volume is correlated with HRQoL in patients with metastatic GEP‐NET. Some 71 patients were included in the study. HRQoL and NET‐specific symptoms were assessed with EORTC QLQ‐C30 and EORTC GI.NET21. A summary score was calculated from the output of the QLQ‐C30. Total somatostatin receptor expressing tumour volume was retrospectively evaluated on somatostatin receptor imaging with positron emission tomography‐computed tomography (^68^Ga‐DOTA‐TATE/TOC PET‐CT) in each patient. Simple and multiple linear regression were used to evaluate the correlation between tumour volume and HRQoL, controlling for potential confounders. No correlation was found between total somatostatin receptor expressing tumour volume and QLQ‐C30 summary score. Weak positive correlations were found between total tumour volume and the specific symptoms dyspnoea, diarrhoea and flushing. To the best of our knowledge, this is the first study to evaluate the association between total somatostatin expressing tumour volume and HRQoL. Our results indicate that, while tumour volume is weakly associated with symptom severity of the carcinoid syndrome, other factors might impact more on overall HRQoL.

## 1 INTRODUCTION

Patients with neuroendocrine tumours of the gastrointestinal tract or pancreas (GEP‐NETs) often present with disseminated disease. Nevertheless, despite an occasional high tumour burden, 5‐year survival often exceeds 50%.^1^ In addition, somatostatin analogues (SSA)^2^, ^3^ and peptide receptor radionuclide therapy (PRRT)^4^, ^5^ further improve progression‐free survival (PFS). However, patients with metastatic GEP‐NET have lower HRQoL than the general population, especially within social‐ and role functioning domains.^6^ The presence of bowel symptoms, especially those associated with social stigma,^7^ fatigue and flushing, are known predictors of poor HRQoL.^8^ Hence, improving health‐related quality of life (HRQoL) is important in patients with metastatic GEP‐NET.

While debulking surgery can be used to control symptoms of NET,^9^, ^10^, ^11^ whether it also leads to improved HRQoL is unclear. In addition, inhibition of tumour hormone secretion by SSA‐analogues was not shown to improve QLQ‐C30 global QoL.^2^ Conversely, PRRT has been reliably shown to improve scores in the QLQ‐C30 domains global QoL, diarrhoea, insomnia and appetite loss,^12^ as well as time to deterioration in other domains.^13^ A recently published review noted that no studies had investigated how disease stage and tumour function affected HRQoL in GEP‐NET patients.^14^ However, one study, not included in the above‐mentioned review, did indeed examine the relationship between tumour burden and HRQoL, and reported a moderate correlation between a nonstandard version of tumour stage and total scores of Norfolk QoL‐NET and the GI.NET21 module.^15^

While standard cross‐sectional imaging is helpful to depict tumour morphology, it does not convey information about expression of somatostatin receptors. For this purpose, in order to adequately assess patients with suspected NET, ^68^Ga‐DOTA‐TATE PET‐CT has been shown to have excellent sensitivity and specificity.^16^ A method has recently been described for measuring somatostatin receptor expressing tumour volume (SRETV) and an estimation of total lesion somatostatin receptor expression (TLSRE) on ^68^Ga‐DOTA‐TATE PET‐CT.^17^ Of these, a strong association was demonstrated between total SRETV and PFS.^18^

In summary, whether tumour volume predicts HRQoL in patients with GEP‐NET is unknown. If tumour volume is associated with HRQoL, decreasing tumour volume might improve HRQoL.

## 2 AIMS

We hypothesised that, possibly through mechanisms of increased hormone levels, increased systemic metabolic demands and increased likelihood of gastrointestinal (GI) tract obstruction, increased tumour volume was associated with more symptoms and lower HRQoL. Therefore, our primary aim was to test whether total somatostatin expressing tumour volume, defined either as ∑SRETV or ∑TLSRE measured on PET‐CT images, was correlated with QLQ‐C30 summary scores in patients with metastatic GEP‐NET.

To explore possible causative pathways of this relationship, our secondary aim was to test whether local tumour volume, that is, total SRETV within each anatomical site, was correlated with specific function scales or specific symptoms as defined by QLQ‐C30 complemented by GI.NET21.

## 3 METHODS

### 3.1 Patients

The patients included in this study were a subgroup of those included in a larger previous study of HRQoL in GEP‐NET. This previous study involved all patients alive on 1 September 2019 in the southern hospital region of Sweden, and whose histopathological diagnosis of well‐differentiated (G1‐G2) GEP‐NET had been established between 1 January 2000 and 31 December 2018. Details of the larger study have been published previously.^7^ The following exclusion criteria were used to select the relevant patient cohort for the present study: patients without evidence of metastatic disease (including lymph node metastasis), NET found incidentally during resection of another cancer, synchronous inoperable colorectal cancer, synchronous inflammatory bowel disease, no ^68^Ga‐DOTA‐TATE/TOC PET‐CT within 1 year before or after the date of answering the questionnaires, and tumour‐modulating treatment (PRRT, surgery, chemotherapy, SIRT, ablation) between ^68^Ga‐DOTA‐TATE/TOC PET‐CT and answering the questionnaires. All patients who gave their written consent and met none of the exclusion criteria were included in the current study. The study was approved by the Swedish Ethical Review Authority (DNR 2019–02378) and undertaken in accordance with the Helsinki Declaration.

### 3.2 Medical records

The following information was gathered from each patient's electronic medical record: age, height, weight, gender, date of diagnosis, primary tumour site, tumour grade and Ki67 at first histopathology, stage, most recent urine 5‐HIAA measurement, most recent chromogranin A, presence of SSA treatment, and previous tumour‐modulating therapy (PRRT, surgery, chemotherapy, SIRT, liver ablation). Other comorbidities were also recorded, and based on these a Charlson comorbidity index (CMI) was calculated for each patient in accordance with methods described elsewhere.^19^

### 3.3 Questionnaires

The patients' HRQoL was evaluated with the cancer‐specific, generic questionnaire EORTC QLQ‐C30,^20^ developed by the European Organisation for Research and Treatment of Cancer. This 30‐item instrument is used to construct one global quality of life scale (QL2) and five function scales: Physical functioning (PF2), role functioning (RF2), emotional functioning (EF), cognitive functioning (CF) and social functioning (SF). It also generates nine symptom scales: fatigue (FA), nausea and vomiting (NV), pain (PA), dyspnoea (DY), insomnia (SL), appetite loss (AP), constipation (CO), diarrhoea (DI) and financial difficulties (FI). The answers from the instrument are transformed into linear scales, with 100 representing optimal function or high quality of life according to the EORTC reference manual.^21^ Conversely, symptom scales are reversed, so that 100 represents maximal symptom burden and 0 an absence of symptoms.

To avoid type 1 errors due to multiple testing of different subscales, a QLQ‐C30 summary score was calculated according to the instructions of its authors.^22^ The summary score includes mean scores of all items except QL2 and FI, and has been shown to have the highest discriminatory power compared to other aggregate models.^22^

While validated and well‐used, the QLQ‐C30 does not cover the specific symptoms of NET. Therefore, a NET‐specific complementary instrument, the EORTC GI.NET21,^23^ was used to generate 10 NET‐specific symptom scales: Endocrine symptoms (ED), gastrointestinal symptoms (GI), treatment‐related symptoms (TR), social functioning NET (SFNET), disease‐related worries (DRW), muscle and bone pain (MBP), sexual functioning (SX), information (INF), body image (BI), weight gain (WG) and weight loss (WL).^23^

### 3.4 PET‐CT protocol

The scans were performed using a Discovery MI or Discovery D690 (GE Healthcare) PET‐CT system. ^68^Ga‐DOTA‐TATE was used for somatostatin receptor imaging until 2019. During 2019 there was a shift in production to ^68^Ga‐DOTA‐TOC at Skåne University Hospital. Both ^68^Ga‐DOTA‐TATE and ^68^Ga‐DOTA‐TOC were prepared according to established techniques.^24^, ^25^, ^26^ Intravenous injection of an activity of 2.0–2.5 MBq/kg (minimum administered activity 100 MBq and maximum 300 MBq) was followed 60 min later by a PET‐CT scan from mid‐thigh to the top of the head, and the PET acquisition time was 3.0 min to 3 min 15 s per bed position, depending on the radiotracer and the PET‐CT system. Time‐of‐flight and point‐spread function correction were used for both PET‐CT systems. Either a low‐dose CT scan or a diagnostic CT was performed simultaneously for attenuation correction and anatomic correlation. If a recent diagnostic CT was available, only a low‐dose CT was acquired during the PET‐CT examination.

### 3.5 Image analyses

If a patient had had more than one ^68^Ga‐DOTA‐TATE/TOC within the timeframe allowed by the inclusion criteria, the most recent scan was chosen in terms of the date the questionnaires were answered. Images were analysed retrospectively by AG and ET. Semi‐automatic segmentation of tumours was performed using the software Hermes (Hermes Medical Solutions). SRETV was defined as tumour volume (measured in millilitres, ml) with uptake higher than 50% of maximum standard uptake value (SUVmax) in a volume of interest (VOI). TLSRE was defined as the product of SRETV and mean SUV (SUVmean) per lesion. Pathological uptake of ^68^Ga‐DOTA‐TATE/TOC was considered significant for tumour segmentation if SUVmax was >3 and did not correspond to physiological uptake. A relatively high normal background uptake in the liver meant that manually drawn VOIs were often needed to avoid physiological uptake, as previously described.^18^ Overlap between tumour volumes was avoided. The sum of all SRETV was calculated within seven separate anatomical sites (liver, pancreas, GI tract, mesenteric lymph nodes, other lymph nodes, skeletal and others). Total tumour volume for each patient was the sum of these values and denoted as ∑SRETV. Corresponding calculations were made for ∑TLSRE.

### 3.6 Statistical analysis

Continuous variables involving EORTC QLQ‐C30 and GI.NET scores are presented using descriptive statistics (mean, median and interquartile range), while categorical variables are presented as frequencies and percentages. As it has been suggested that a score below 50 for QoL/function scales or above 50 for symptom scales denotes significantly impaired HRQoL, the number of patients with scores above or below 50, respectively, was counted for each function/symptom scale. To describe tumour distribution in the cohort, median SRETV was calculated for all patients with a tumour in each respective anatomical site. Since the SRETV in the liver was disproportionately higher than in the other anatomical sites, and in order to quantify the distribution of tumour volume in each patient further, a ratio between SRETV in the liver (SRETV_liver_) and ∑SRETV was calculated for each patient.

Since the distributions of ∑SRETV and ∑TLSRE were found to be highly skewed (skewness values of 3.3 and 2.8, respectively), they were transformed to their natural logarithms, log∑SRETV and log∑TLSRE. Simple linear regression was then performed between the dependent variable QLQ‐C30 summary score and log∑SRETV. To control for possible confounders, we performed multiple linear regression between QLQ‐C30 and log∑SRETV with adjustment for age, Charlson CMI and SSA treatment. To explore whether previous major surgery using Whipple's procedure would interfere with the results, a sensitivity analysis was made which excluded these patients. Another sensitivity analysis was carried out which excluded all patients with ^68^Ga‐DOTA‐TATE/TOC more than 6 months before or after answering the questionnaire. In addition, subgroup analysis of patients with grades 1 and 2 and diagnosis less than or more than 4 years before undergoing the scan was made. A second sensitivity analysis was performed excluding non small intestine NET patients and those with recent (<6 months) tumour directed treatment. The same procedure was then performed with log∑TLSRE as the independent variable.

To analyse the secondary aim, a correlation table was made with Pearson's correlation coefficients between logSRETV in each anatomical site and each function scale/symptom scale from EORTC QLQ‐C30 and GI.NET21. As suggested in previous literature,^27^ a cutoff of *r* > 0.2 was chosen to separate weak correlation from no correlation.

## 4 RESULTS

Between 1 January 2000 and 31 December 2018, 806 unique patients received a histopathological diagnosis of GEP‐NET in the Skåne healthcare region. Of these, 561 patients were excluded from the analysis by one exclusion criterion or more, as follows: where NET had been found incidentally during the resection of another cancer, or if there was synchronous inoperable colorectal cancer, synchronous inflammatory bowel disease or a localised tumour where endoscopic excision or appendectomy had sufficed as treatment. Between 2 September 2019 and 27 September 2019, the remaining 245 patients were invited by normal post to participate in the study. Of these, three declined to participate, and 10 had to be excluded as they had emigrated or their address was unknown. A further 67 patients did not respond. The remaining 165 patients returned the completed instruments and signed consent forms, resulting in a response rate of 67%. For the present study, a further 94 patients were excluded: some 92 due to non‐metastatic disease, one due to chemotherapy between scan and questionnaire and one subsequently declining to participate. The final cohort included 71 patients.

### 4.1 Patient characteristics

Patient characteristics are displayed in Tables 1 and 2. Mean (standard deviation, SD) age was 69.8 (9.8) years, and 42 (60%) patients were male. Mean (SD) time since diagnosis was 5.5 (3.7) years. Some 58 patients (82%) were receiving SSA treatment and 32 patients (45%) had elevated levels of U 5‐HIAA. Some 15 patients (21%) had stage III disease, the remaining 56 patients (79%) had stage IV disease. Mean QLQ‐C30 summary score was high in the cohort, with a value of 82.3 (SD 14.4). However, within areas of social, emotional and role function, 13, 10 and 10 patients respectively had very low function, with scores ≤50. Severe symptomatology, that is, scores ≥50, was most common with diarrhoea (*n* = 24), disease‐related worries (*n* = 25) and muscle/bone pain (*n* = 16). Some 61 patients (86%) had small intestine as their primary tumour site. Of the 71 patients in the cohort, some 42 patients had undergone small bowel resection, and 23 patients right‐sided hemicolectomy or ileocaecal resection.

**TABLE 1:** Patient characteristics

	Mean (SD)	Median (IQR)	*n* with ≤50 (%)^a^	Missing
Age (years)	69.8 (9.8)	70 (62–78)		0
Weight (kg)	76.3 (18.4)	75 (66–85)		0
Height (cm)	170.4 (22.3)	173 (167–180)		0
Body mass index (BMI)	25.5 (5.6)	25 (21.9–27.4)		0
Charlson comorbidity index (CMI)	3.8 (2.1)	3 (2–6)		0
Years since diagnosis	5.5 (3.7)	4.3 (2.8–7.1		0
Days between questionnaire and scan	119 (82)	106 (47–176)		0
QLQ‐C30 and GI.NET21				
QLQC30 summary score	82.3	84.1 (76.1–92.4)	2 (2.8)	0
Global quality of life QL2	71.2	21.8 (50.0–75.0)	18 (25.4)	0
Physical function PF2	85.2	93.3 (73.3–100)	4 (5.6)	0
Role function RF2	83.6	100 (66.7–100)	10 (14.1)	0
Emotional function EF	79.8	83.3 (66.7–100)	10 (14.1)	0
Cognitive function CF	87.3	83.3 (66.7–100)	2 (2.8)	0
Social function SF	80.8	83.3 (66.7–100)	13 (18.3)	0
Fatigue FA	28.3	22.2 (11.1–44.4)	13 (18.3)	0
Nausea and vomiting NV	4.2	0 (0–0)	1 (1.4)	0
Pain PA	18.5	0 (0–33.3)	12 (16.9)	0
Dyspnoea DY	20.2	0 (0–33.3)	9 (12.7)	0
Sleep disturbance SL	25.4	33.3 (0–33.3)	11 (15.5)	0
Appetite loss AP	7.5	0 (0–0)	2 (2.8)	0
Constipation CO	6.1	0 (0–0)	2 (2.8)	0
Diarrhoea DI	36.6	33.3 (0–66.7)	24 (33.8)	0
Endocrine dysfunction ED	12.3	11.1 (0–22.2)	1 (1.4)	0
Financial difficulties FI	6.6	0 (0–0)	3 (4.2)	0
Gastrointestinal GI	20.4	13.3 (6.7–33.3)	5 (7.0)	0
Treatment‐related TR	17.2	16.7 (0–33.3)	4 (5.6)	12
Disease‐related worries DRW	43.6	33.3 (22.2–66.7)	25 (35.2)	0
Social function NET SFNET	27.3	22.2 (0–44.4)	17 (23.9)	0
Weight loss WL	18.3	0 (0–33.3)	13 (18.3)	0
Weight gain WG	3.9	0 (0–0)	4 (5.8)	2
Muscle and bone pain MBP	26.1	33.3 (0–33.3)	16 (23.2)	2
Information INF	8.6	0 (0–0)	6 (8.6)	1
Sexual dysfunction SX	28.5	0 (0–50)	12 (25.0)	23

Abbreviations: IQR, interquartile range; SD, standard deviation.

^a^ For symptom scores ≥50.

**TABLE 2:** Patient characteristics

	*n* (%)	Missing
Male gender	42 (59.1)	0
Grade 1	42 (60.0)	2
Grade 2	26 (37.1)	2
Chromogranin A ≥ 2	33 (46.5)	1
Urine 5‐HIAA >30	32 (45.1)	3
SSA treatment	58 (81.7)	0
Previous PRRT	8 (11.3)	0
Previous chemotherapy	5 (7.0)	0
Previous tumour surgery		
Pancreaticoduodenectomy (Whipple)	6 (8.5)	0
Distal pancreatic resection	4 (5.6)	0
Small bowel resection	42 (59.1)	0
Right‐sided hemicolectomy or ileocaecal resection	23 (32.4)	0
Other colorectal resection	1 (1.4)	0
Splenectomy	1 (1.4)	0
Liver resection	3 (4.2)	0
Primary tumour site		
Small intestine	61 (85.9)	0
Pancreas	9 (12.7)	0
Duodenum	1 (1.4)	0
Disease stage		
III	15 (21)	
IV	56 (79)	

Tumour distribution	*n* (%)	Median ∑SRETV, ml (IQR)
Liver	40 (56.3)	18.8 (10.6–112.7)
Pancreas	15 (21.1)	1.7 (0.7–3‐7)
Mesenteric lymph nodes	36 (50.7)	1.6 (0.7–4‐3)
GI tract	9 (12.7)	1.4 (1.0–3.3)
Other lymph nodes	31 (43.7)	1.4 (0.5–4.8)
Skeletal	17 (23.9)	1.2 (0.8–6.9)
Other	13 (18.3)	2.4 (1.3–4.2)
Total	71 (100)	10 (2–45)

*Note*: Tumour surgeries not exclusive, that is, some patients have undergone more than one type of surgery. Tumour distribution denotes count and frequencies of patients with tumour in each anatomic site.

Abbreviations: ∑SRETV, total somatostatin receptor expressing tumour volume; GI, gastrointestinal; IQR, interquartile range; PRRT, peptide receptor radionuclide therapy; SSA, somatostatin analogue; Urine 5‐HIAA, urine 5‐hydroxyindoleacetic acid.

### 4.2 Tumour volume distribution

Median ∑SRETV was 9.6 (interquartile range [IQR] 1.7–45.4) ml. The maximum value was 539 ml, indicating highly skewed data. Some 40 patients (56%) had visible liver metastases of NET on ^68^Ga‐DOTA‐TATE/TOC. Of these, the median SRETV was 18.8 ml. Meanwhile, there was considerable variability of SRETV in the liver, with an IQR of 10.6–112.7 ml. In comparison, median SRETV in patients with evidence of NET in the mesenteric lymph nodes or NET in the GI tract was only 1.6 and 1.4 ml, respectively. For the 40 patients with liver metastases, the median ratio of SRETV_liver_/∑SRETV was 0.93 (IQR 0.66–0.99). This suggests that for a significant number of patients in the cohort, tumour volume in the liver made up a substantial part of total tumour volume. For details, see Table 2.

### 4.3 Health‐related quality of life and tumour volume

**FIGURE 1:**
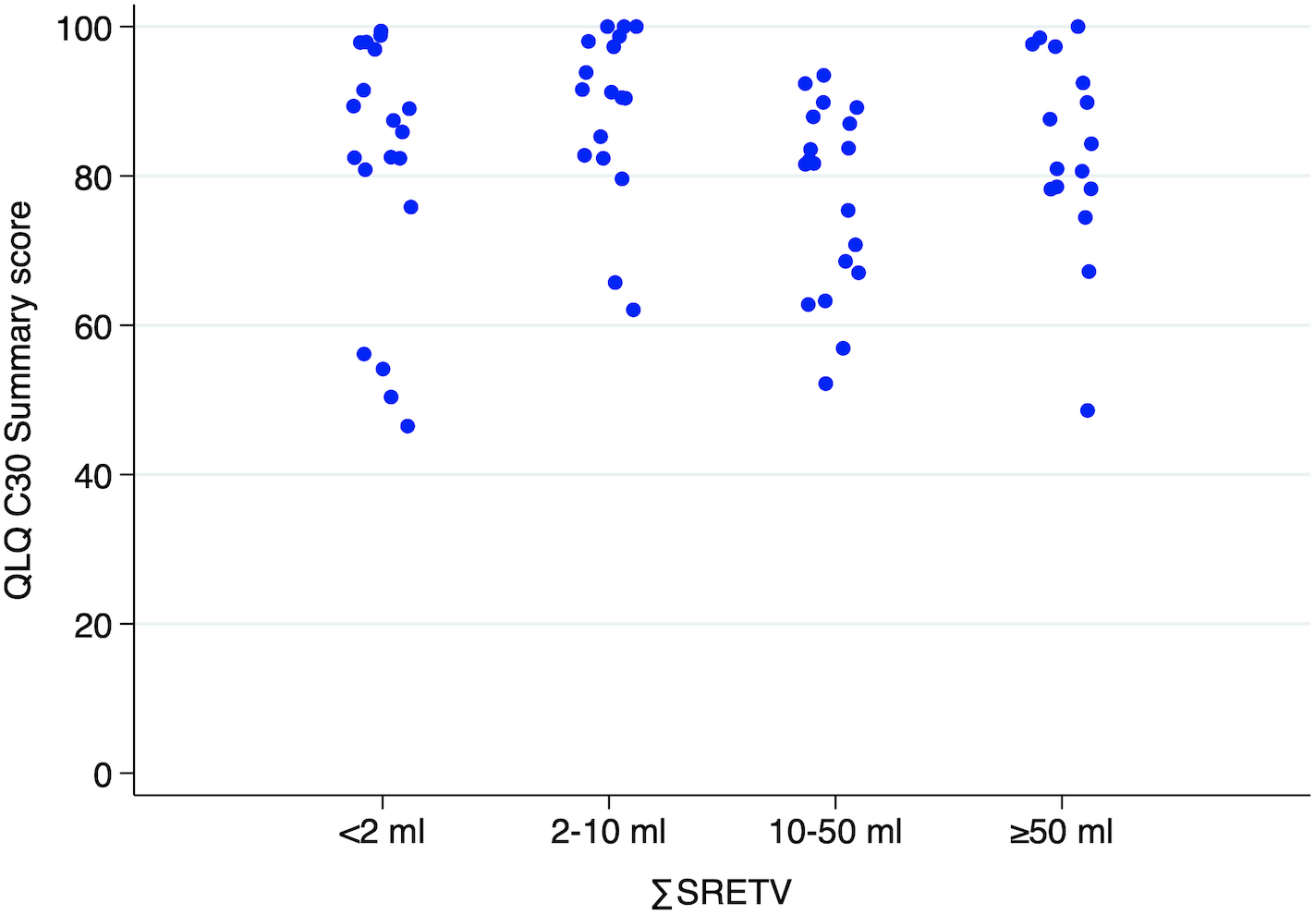
Scatterplot of individual QLQ‐C30 summary scores with patients grouped into approximate quartiles of total SRETV

Tables 3 and 4 show the results of the primary analysis. Simple linear regression between log∑SRETV and QLQ‐C30 summary score showed no correlation between the variables, with a beta‐coefficient of 0.13 (95% CI: −1.59‐1.85, *p* = .88). Multiple linear regression with adjustment for age, Charlson CMI and treatment with SSA showed a similarly nonsignificant result, with a beta‐coefficient of 0.39 (95% CI: −1.49‐2.26, *p* = .683). Sensitivity analyses which excluded the six patients who had undergone pancreaticoduodenectomy, or the 15 patients with more than 6 months between answering the questionnaire and undergoing ^68^Ga‐DOTA‐TATE/TOC, did not change these results. Subgroup analysis of patients according to grade and recent diagnosis showed the same results (Table 4). Sensitivity analysis with exclusion of non si‐NET patients and three patients with recent tumour directed therapy (3 patients undergoing PRRT) did not alter the results (see Table S1). A corresponding analysis for log∑TLSRE and QLQ‐C30 summary score did not show a statistically significant correlation. For illustrative purposes, ∑SRETV was divided into approximate quartiles and presented with a QLQ‐C30 summary score on the y‐axis (Figure 1).

**TABLE 3:** Main results

	1. Simple linear regression	2. Multiple linear regression (age, SSA treatment, Charlson CMI)	3. Exclusion of post‐Whipple patients	4. Exclusion of patients with >6 months between questionnaire and scan	Both 3 and 4	2,3 and 4
*n*	71	71	65	56	53	53
∑SRETV (beta‐coefficient, 95% CI)	0.1 (−1.6–1.9)	0.4 (−1.5–2.3)	0.05 (−1.8–1.9)	−1.0 (−3.0–1.0)	−1.2 (−3.4–0.9)	−1.0 (−3.3–1.3)
∑TLSRE (beta‐coefficient, 95% CI)	0.06 (−1.5–1.6)	0.3 (−1.4–1.9)	0.1 (−1.5–1.7)	−0.7 (−2.5–1.1)	−0.8 (−2.7–1.1)	−0.6 (−2.6–1.4)

Abbreviations: ∑SRETV, total somatostatin receptor expressing tumour volume; ∑TLSRE, total lesion somatostatin receptor expression; 95% CI, 95 percent confidence interval; CMI, comorbidity index; SSA, somatostatin analogue.

**TABLE 4:** Subgroup analysis

	Grade 1		Grade 2	
	Simple linear regression	Multiple linear regression (age, SSA treatment, Charlson CMI)	Simple linear regression	Multiple linear regression (age, SSA treatment, Charlson CMI)
*n*	42	42	26	26
∑SRETV (beta‐coefficient, 95% CI)	1.7 (−0.4–3.9)	2.0 (−0.4–4.3)	−2.2 (−5.2–0.8)	−1.9 (−5.2–1.4)
∑TLSRE (beta‐coefficient, 95% CI)	1.4 (−0.5–3.4)	1.7 (−0.5–3.9)	−1.8 (−4.5–0.9)	−1.5 (−4.3–1.4)

	Diagnosis <4 years		Diagnosis >4 years	
	Simple linear regression	Multiple linear regression (age, SSA treatment, Charlson CMI)	Simple linear regression	Multiple linear regression (age, SSA treatment, Charlson CMI)
*n*	32	32	39	39
∑SRETV (beta‐coefficient, 95% CI)	1.2 (−1.7–4.2)	0.7 (−3.8–4.2)	−1.0 (−3.0–1.1)	−0.5 (−2.6–1.6)
∑TLSRE (beta‐coefficient, 95% CI)	0.9 (−1.7–3.5)	0.4 (−2.7–3.5)	−0.8 (−2.6–1.0)	−0.4 (−2.3–1.4)

Abbreviations: ∑SRETV, total somatostatin receptor expressing tumour volume; ∑TLSRE, total lesion somatostatin receptor expression; 95% CI, 95 percent confidence interval; CMI, comorbidity index; SSA, somatostatin analogue.

Table 5 shows the results of the secondary analysis. The only function scales that correlated with SRETV in any anatomic site were role function (RF2) and emotional function (EF). Both were correlated with SRETV in the mesenteric lymph nodes, with *r* = 0.24 and *r* = 0.21, respectively. The symptoms which bothered the most patients, diarrhoea (DI) and muscle/bone pain (MBP), were correlated with increased SRETV in different anatomical sites: DI was most correlated with SRETV in the GI tract (*r* = 0.31), SRETV in the liver (*r* = 0.28), SRETV in other lymph nodes (*r* = 0.20) and SRETV at other unspecified sites (*r* = 0.25). Conversely, MBP was only weakly correlated with SRETV in the mesenteric lymph nodes (*r* = 0.21). Disease‐related worries (DRW) were not correlated with SRETV in any anatomical site. Total tumour volume, ∑SRETV, showed weak positive correlation with dyspnoea (*r* = 0.21), diarrhoea (*r* = 0.23), endocrine dysfunction (*r* = 0.33) and information (*r* = 0.27), and weak negative correlation with weight gain (−0.27).

**TABLE 5:** Correlation table, tumour volume versus function‐ and symptom scales

	Liver	Pancreas	GI tract	Mesenteric lymph nodes	Other lymph nodes	Skeletal	Other	Total
Global QoL QL2	−0.081	0.222	0.085	0.026	−0.013	0.104	0.0317	−0.017
Physical function PF2	−0.010	−0.007	0.044	0.057	0.105	−0.097	0.110	0.043
Role function RF2	0.046	0.130	0.065	0.244	0.178	0.138	0.039	0.141
Emotional function EF	−0.007	0.165	0.134	0.208	0.193	0.009	0.012	0.112
Cognitive function CF	−0.147	0.015	0.133	0.048	−0.043	−0.101	0.023	−0.119
Social function SF	0.022	0.159	0.035	0.086	0.098	0.019	−0.040	0.073
Fatigue FA	0.096	−0.155	0.039	0.051	−0.108	0.007	0.036	0.036
Nausea and vomiting NV	0.104	−0.061	−0.003	−0.078	−0.259	−0.186	−0.187	0.000
Pain PA	−0.102	−0.201	0.042	−0.031	−0.191	−0.127	0.118	−0.171
Dyspnoea DY	0.299	−0.103	0.036	0.028	0.083	0.043	−0.038	0.207
Sleep disturbance SL	0.084	−0.208	0.058	0.034	−0.147	−0.055	0.190	0.004
Appetite loss AP	0.241	−0.038	0.009	−0.038	−0.270	−0.175	0.160	0.181
Constipation CO	0.011	−0.118	−0.083	−0.100	−0.140	0.002	−0.083	−0.073
Diarrhoea DI	0.280	−0.085	0.310	0.097	0.201	−0.079	0.251	0.230
Endocrine dysfunction ED	0.345	−0.033	0.259	0.102	0.011	−0.029	0.223	0.325
Gastrointestinal GI	0.082	−0.090	0.018	0.181	0.115	−0.139	0.020	0.096
Treatment‐related TR	−0.142	−0.200	0.147	0.197	0.030	−0.339	0.109	−0.120
Disease‐related worries DRW	−0.086	−0.134	−0.176	−0.072	0.051	0.082	−0.078	−0.154
Social function NET SFNET	0.082	−0.052	−0.083	0.004	0.016	0.034	0.071	0.078
Weight loss WL	0.050	−0.159	0.050	0.194	0.195	−0.047	0.169	0.088
Weight gain WG	−0.195	−0.004	0.037	−0.284	−0.346	−0.342	0.008	−0.229
Muscle and bone pain MBP	0.103	−0.087	0.167	0.203	0.008	−0.083	0.088	0.078
Information INF	0.303	0.007	−0.078	−0.044	0.307	0.186	0.151	0.267
Sexual dysfunction SX	0.173	−0.094	0.111	0.166	0.186	0.283	0.319	0.113
*n* with tumour on each site	40	15	9	36	31	17	13	71

*Note*: Correlation table with Pearson's correlation coefficients (*r*‐values).

## 5 DISCUSSION

In the present study, we could not confirm that somatostatin expressing tumour volume on ^68^Ga‐DOTA‐TATE/TOC in patients with metastatic GEP‐NET, measured as either ∑SRETV or ∑TLSRE, was associated with HRQoL. Linear regression, with both a simple unadjusted model and a multiple adjusted model, indicated no correlation between either ∑SRETV or ∑TLSRE and EORTC QLQ‐C30 summary scores. Subgroup analysis and repeated sensitivity analysis which excluded possible confounders did not alter these results. However, results from the secondary analysis could indicate a correlation between ∑SRETV and the specific symptoms of diarrhoea, flushing and dyspnoea. To our knowledge, these findings have not previously been reported.

It might seem intuitive that increased tumour volume would lead to a proportionate decrease in HRQoL through the causal pathway where tumour volume causes symptoms, which causes decreased HRQoL. Our findings contradict this notion. A potential explanation may be that the disease in most patients in our cohort was stable, and most symptoms were not new or worsening. Adaptation level theory^28^ suggests that most people can adapt to unwanted events over time.^29^ Effects such as this could perhaps also explain how our findings are consistent with findings showing a prolonged time to deterioration of QoL in patients with advanced progressive midgut NET who are receiving peptide receptor radionuclide therapy (PRRT).^13^ In this latter study, patients rated their HRQoL regularly, and as their disease progressed, HRQoL decreased correspondingly. Presumably by inhibiting disease progress, so that the volume of each patient's tumour was lower than what it would have been without treatment, PRRT effectively blocked any deterioration of symptoms and a resulting decrease in HRQoL. Psychological mechanisms for coping and adaptation may explain why this difference is not discernible in studying an entire cohort with a cross‐sectional design, as in our study. In parallel to this, a substantial part of our cohort was suffering from severe disease‐related worries unrelated to tumour volume, suggesting that coping strategies are more important to HRQoL than the extent of the disease itself. It could also be hypothesised that progressive disease is a more important factor in explaining decreased HRQoL than the volume of the tumour itself. This is supported by the findings of Khan et al., who not only reported significantly lower HRQoL in patients with progressive disease before treatment with PRRT compared to patients with stable disease, but also increased HRQoL after PRRT for all patients, regardless of treatment outcome.^12^

HRQoL is a multifaceted concept which consists of patient‐reported somatic, psychological and social aspects.^30^ Therefore, it could be argued that the QLQ‐C30 summary score is too general to evaluate the relationship between HRQoL and tumour volume. Accordingly, the findings of our secondary analysis, which showed an association between ∑SRETV, SRETV_liver_ and some symptoms of carcinoid syndrome (diarrhoea, flushing, dyspnoea), might indicate that although tumour volume does not affect HRQoL in a general sense, it still affects the extent of symptom severity. Since total SRETV in the majority of patients in this study consists mostly of liver metastases, this is consistent with the general biological behaviour of functioning NETs, which require metastasis to the liver for symptoms of carcinoid syndrome to develop. It is also consistent with the findings of Tirosh et al., which indicate a positive correlation between tumour volume assessed by ^68^Ga‐DOTA‐TATE and 24‐h urine 5‐hydroxyindoleacetic acid (U 5‐HIAA) in patients with small‐intestinal NET (*r* = 0.7, *p* < .001).^31^

The findings of Vinik et al.,^15^ which indicate a moderate correlation between total scores of EORTC QLQ‐C30 + GI.NET21 and tumour burden graded from 1 to 6, also support the fact that tumour burden affects symptom severity. Conversely, in our study, no correlation was found between total tumour volume and each of the function scales PF2, RF, EF, CF, SF or global quality of health, QL2. This could suggest that, while total SRETV might affect symptoms, possibly through psychological mechanisms, it does not significantly affect patients' ability to lead their lives.

One strength of our study is its population‐based design, which should minimise selection bias. Equally, compared to previous studies measuring tumour burden, the method of measuring SRETV and TLSRE described here is more detailed and, in terms of total metabolic demand and total hormone‐producing ability, biologically more coherent.

Meanwhile, some limitations should be noted in terms of measurements of tumour volume. At the present moment, no validated method is available for measuring SRETV, but it can be measured in a number of ways. We chose a method that has been described before, and which has also been shown to have potential prognostic value.^18^ Also, the method of delineation with 50% of SUVmax had limitations, so that when the tumours were large and heterogeneous, with both necrotic parts and parts with high uptake, it is possible that not all metabolic tumour volume was included. This is consistent with the fact that methods of fixed relative threshold‐based measurements of tumour volume have been shown to have limitations with ^18^F‐FDG PET‐CT, where tumours with necrotic cores and lesions with low uptake relative to the background could be under‐ or overestimated.^32^ The different radiotracers used in the study, ^68^Ga‐DOTA‐TATE/TOC might also have a modest impact on an individual basis. Nevertheless, primarily because all tumours were grade 1 or grade 2 and only one patient had Ki67 above 15%, we do not judge these limitations to be significant enough to affect the overall outcome of the study. Other limitations include the cross‐sectional, retrospective design, the small sample size and the fact that participation in the study was voluntary. The latter could have led to a problem of nonrandom missing data due to selection bias, by including more (or fewer) healthy patients than the general population of patients with GEP‐NET. Since medical records were inaccessible for the patients who were not included in the study, there was no opportunity to investigate whether this had been an issue in the present cohort.

In summary, although a weak correlation was noted between some symptoms of carcinoid syndrome and tumour volume, this study showed no correlation between overall HRQoL and total tumour volume in patients with metastatic GEP‐NET. Our results could point to the importance of coping strategies in patients with incurable malignant disease, and may provide hypotheses for further research in this area.

## CONFLICT OF INTEREST

The senior author has received research grants from IPSEN, and consultancy fees from Medtronic, outside this study. AS has received speaker and consultancy fees from IPSEN, Novartis, Sam Nordic and Spago Nanomedical, none of which are related to this study. The other authors declare no conflicts of interest.

## AUTHOR CONTRIBUTIONS

**Håkan Ohlsson:** Conceptualization; data curation; formal analysis; funding acquisition; investigation; methodology; project administration; resources; software; validation; visualization; writing – original draft; writing – review and editing. **Anni Gålne:** Conceptualization; data curation; formal analysis; funding acquisition; investigation; methodology; project administration; resources; software; validation; writing – original draft; writing – review and editing. **Elin Trägårdh:** Conceptualization; data curation; funding acquisition; investigation; methodology; resources; software; supervision; writing – review and editing. **Marlene Malmström:** Conceptualization; methodology; supervision; validation; writing – review and editing. **Anna Sundlöv:** Conceptualization; methodology; supervision; validation; writing – review and editing. **Martin Almquist:** Conceptualization; formal analysis; funding acquisition; methodology; resources; software; supervision; writing – review and editing.

### PEER REVIEW

The peer review history for this article is available at https://publons.com/publon/10.1111/jne.13139.

## Supporting information


**Table S1.** Supplement.

## Data Availability

The data that support the findings of this study are available on request from the corresponding author. The data are not publicly available due to privacy or ethical restrictions.
