# Supplementary material for: Relationship between somatostatin receptor expressing tumour volume and health‐related quality of life in patients with metastatic GEP‐NET
Source: J Neuroendocrinol. 2022 Apr 29;34(6):e13139. doi: 10.1111/jne.13139 (PMC9285792; doi:10.1111/jne.13139)
Supplement: Supplementary file 1 — Table S1. Supplement. [file JNE-34-0-s001.pdf]

Supplement

Sensitivity analysis 2						
	5. Only si-NET, simple linear regression	6. Only si-NET with multiple linear regression (age, SSA treatment, Charlson CMI)	7. Only si-NET and exclusion of patients with >6 months between questionnaire and scan.	8. Only si-NET with exclusion of all patients with recent treatment.	Both 7 and 8	6,7 and 8
**n**	61	61	49	58	46	46
**∑SRETV (beta-coefficient, 95% CI)**	0.02 (-1.9-1.9)	0.3 (-1.8-2.4)	-1.1 (-3.4-1.3)	0.5(-1.3-2.3)	-0.7 (-2.8-1.5)	-0.4 (-2.7-1.9)
**∑TLSRE (beta-coefficient, 95% CI)**	0.11 (-1.5-1.6)	0.4 (-1.5-2.2)	-0.7 (-2.8-1.4)	0.4(-1.2-2.1)	-0.4 (-2.3-1.5)	-0.3 (-2.3-1.7)

Legend sensitivity analysis: ∑SRETV = total somatostatin receptor expressing tumour volume. ∑TLSRE = total lesion somatostatin receptor expression. 95% CI = 95 percent confidence interval.

For Peer Review Only
